# EEL-GA: An Evolutionary Clustering Framework for Energy-Efficient 3D Wireless Sensor Networks in Smart Forestry

**DOI:** 10.3390/s25175250

**Published:** 2025-08-23

**Authors:** Faryal Batool, Kamran Ali, Aboubaker Lasebae, David Windridge, Anum Kiyani

**Affiliations:** Department of Computer Science, Middlesex University, London NW4 4BT, UK; k.ali@mdx.ac.uk (K.A.); a.lasebae@mdx.ac.uk (A.L.); d.windridge@mdx.ac.uk (D.W.); a.kiyani@mdx.ac.uk (A.K.)

**Keywords:** 3D wireless sensor networks, EEL-GA, genetic algorithm, LEACH, residual energy, energy-aware clustering, forest monitoring, coverage optimization, cluster head selection

## Abstract

Wireless Sensor Networks (WSNs) are very important for monitoring complex 3D environments like forests, where energy efficiency and reliable communication are critical. This paper presents EEL-GA, an Energy Efficient LEACH-based clustering protocol optimized using a Genetic Algorithm. Cluster head (CH) selection is guided by a dual-metric fitness function combining residual energy and intra-cluster distance. EEL-GA is evaluated against EEL variants using Particle Swarm Optimization (PSO), Differential Evolution (DE), and the Artificial Bee Colony (ABC) across key performance metrics, including energy efficiency, packet delivery, and cluster lifetime. Simulations using real environmental data confirm EEL-GA’s superiority in sustaining energy, minimizing delay, and improving network stability, making it ideal for smart forestry and mission-critical WSN deployments. The model also incorporates environmental dynamics, such as temperature and humidity, enhancing its robustness in real-world applications. These findings support EEL-GA as a scalable, adaptive solution for future energy-aware 3D WSN frameworks.

## 1. Introduction

Many applications, such as environmental monitoring, disaster response, and environmental preservation nowadays increasingly rely on Wireless Sensor Networks (WSNs) for efficient data collection and communication [1]. Wireless Sensor Networks (WSNs) are composed of sensor nodes distributed across different locations to collect, process, and send data to Base Stations (BSs). Deploying these networks in complex 3D forest environments can be challenging due to issues with energy efficiency, reliable communication, and adapting to the terrain [2].

Smart forestry uses Wireless Sensor Networks (WSNs) to monitor environmental conditions continuously. These networks include sensor nodes placed in different areas to send information about factors like soil moisture, temperature, and humidity [3,4]. The deployment of these sensors enables real-time data collection, which is vital for understanding forest dynamics and health. By utilizing IoT technology, these networks can connect to the cloud for storage and analysis, providing stakeholders with a comprehensive view of operational conditions [5]. This integration facilitates proactive management strategies, allowing forest managers to respond to environmental changes or potential threats, such as pests or diseases, expeditiously.

In smart forestry, WSNs serve as a backbone for real-time monitoring of environmental parameters, including temperature, humidity, and soil moisture, which play a crucial role in forest management and conservation strategies [6]. Given the remote and often challenging conditions of forest environments, energy efficiency in these networks is paramount. Energy limitations significantly impact network longevity, requiring innovative approaches to clustering and data transmission.

This framework can be implemented using a tiered approach that segments the forestry area into 3D spatial clusters, enabling localized data aggregation and processing to minimize the energy overhead associated with long-range data transmission [7]. Each cluster can dynamically reconfigure itself based on sensor states using adaptive learning-based strategies, effectively distributing communication load and mitigating energy bottlenecks [6]. In practical deployments, this may involve sensor nodes equipped with multi-tier clustering protocols, such as Region-based Hierarchical Clustering for Efficient Routing (RHCER), capable of handling large-scale forestry data while accounting for node-level energy constraints in routing decisions [8]. This evolutionary clustering approach enhances WSN performance in several ways: it extends network lifetime by balancing load during cluster head selection [9,10], improves scalability by adapting to larger deployment areas using evolutionary algorithms [11], and boosts energy efficiency through optimal routing and intra-cluster communication [12,13]. Studies have reported lifespan improvements of approximately 30–46% through metaheuristic clustering protocols involving optimized cluster head selection and routing algorithms [14]. Additionally, this architecture supports real-time environmental monitoring, which is vital for smart forestry management and rapid responses to ecological changes [8,9].

Traditional WSN clustering protocols, such as LEACH, are primarily designed for two-dimensional (2D) environments and often struggle to adapt to 3D topologies, where elevation changes, irregular terrain, and dense foliage significantly impact communication and energy dissipation [15,16]. In forest environments, issues like uneven node placement, long transmission distances, and high path loss frequently lead to rapid energy depletion and network partitioning [17]. To address these challenges, recent advancements have applied evolutionary approach algorithms, including Particle Swarm Optimization (PSO), Genetic Algorithms (GAs), and Differential Evolution (DE) to improve clustering performance and network lifespan [18,19]. While promising, many of these approaches assume static environmental conditions and overlook critical factors, such as residual energy dynamics and intra-cluster communication overhead [20]. This study proposes an enhanced Energy-Efficient LEACH protocol integrated with a Genetic Algorithm (EEL-GA) tailored for 3D WSN deployments. Our model dynamically selects cluster heads (CHs) based on real-time residual energy and 3D intra-cluster distances, guided by a dual-metric fitness function that balances spatial compactness and energy fairness. Unlike conventional clustering models, EEL-GA adapts to topological fluctuations and environmental variations, significantly improving energy conservation and routing efficiency.

In order to contextualize our approach, we consider a use case involving a forested national park prone to seasonal wildfires and illegal logging. In such areas, WSNs can be deployed on tree trunks, the forest canopy, and ground surfaces to monitor environmental conditions and detect unusual activities. Sensors placed at different altitudes report varying levels of signal strength, delay, and energy consumption due to obstruction by dense foliage and terrain elevation. To ensure real-time monitoring and long-term network sustainability in such dynamic conditions, an intelligent, adaptive clustering mechanism becomes essential. This scenario reflects the operational needs that our EEL-GA protocol is designed to address. Moreover, the complex environmental variability and node placement heterogeneity in forest terrains make it necessary to evaluate WSN protocols through simulation before real-world deployment. Our simulation model simulates a realistic forest area using terrain data and empirical measurements. This method allows us to analyze the impact of energy dynamics, cluster configurations, and routing strategies under controlled yet representative conditions.

To evaluate the proposed method, we utilize an environmental dataset collected as part of a research project in the Ziarat Forest region of Balochistan, Pakistan. This dataset includes temporal readings of temperature, humidity, wind speed, and rainfall collected via physical sensor deployments across the forest terrain. These environmental factors were integrated into the simulation model to affect node energy consumption, communication quality, and decision-making under dynamic conditions.

In summary, the deployment of WSNs in complex forest environments faces key challenges: (i) traditional clustering protocols are poorly suited to 3D terrains; (ii) random CH selection contributes to unbalanced energy drain; (iii) environmental influences on communication and power usage are often neglected; and (iv) static routing fails to sustain communication in dynamic topologies. The proposed EEL-GA addresses these issues by introducing an energy-aware, topology-adaptive clustering strategy supported by empirical data and multi-objective optimization.

To address these gaps, this work contributes the following:

i.   A 3D WSN Simulation: A simulation environment that models sensor deployment in a 3D forest topology using real-world environmental data.
ii.  EEL-GA Clustering Protocol: A hybrid clustering strategy combining LEACH with a Genetic Algorithm to adaptively select energy-aware CHs.
iii. Dual-Metric Fitness Function: A novel fitness model that evaluates CH candidates based on residual energy and intra-cluster distance.
iv. Comparative Evaluation: Benchmarking against EEL-PSO, EEL-DE, and EEL-ABC to validate performance on metrics, including residual energy, cluster lifetime, and latency.
v.  Deployment Suitability: Demonstrated effectiveness of the protocol in smart forestry use cases requiring long-term, energy-efficient monitoring.

## 2. Related Work

Energy-efficient clustering remains a pivotal concern in WSNs, particularly under challenging 3D deployment conditions where energy drainage is accelerated due to longer inter-node distances and irregular terrain. Several optimization strategies have emerged to address these challenges by enhancing energy balance, extending network lifetime, and improving spatial coverage [21,22].

To reduce communication overhead and prolong network lifetime, early clustering techniques, such as LEACH (Low-Energy Adaptive Clustering Hierarchy), were introduced [15]. LEACH periodically rotates the role of CHs to evenly distribute energy consumption among nodes. While effective in simple deployments, its probabilistic CH selection often leads to uneven energy depletion and reduced efficiency—particularly in dense or topologically irregular networks [23,24]. The original LEACH protocol suffers from several well-known limitations, including random cluster head (CH) selection, uneven CH distribution, single-hop communication, and limited adaptability to network dynamics [25,26]. According to Ref. [27] propose the MCH-EOR algorithm, which uses a multi-objective fitness function based on residual energy and inter-node distances to optimize cluster-head selection and improve network lifetime and throughput in WSNs, utilizing residual energy and node location data to optimize CH selection. Multi-hop LEACH protocols [28,29] reduce the communication burden for distant CHs by introducing relay nodes. Adaptive extensions, like E-LEACH, dynamically adjust CH roles to balance load and prolong network life [30]. Optimization-based variants incorporate metaheuristic algorithms, such as GAs [31,32], bat algorithms [33], firefly PSO [34], and fuzzy–PSO [35], to improve clustering decisions. PSO-based variants, like [19], dynamically adjust CH roles using spatial and energy-based heuristics. Clustering strategies using K-means, GWO, and Gaussian models have also been studied [36,37]. Additionally, Differential Evolution (DE) [38,39] and Artificial Bee Colony (ABC) [40] algorithms have shown promise in improving CH diversity and minimizing node-level energy usage. These studies highlight the effectiveness of evolutionary methods in improving energy efficiency and network performance.

While most existing studies focus on flat, simulated 2D environments, fewer works address realistic 3D deployments in natural terrains. Notably, ref. [41] presented a comparative study on clustering protocols in synthetic forest environments, showing how terrain and node density affect energy use. Kumar et al. [42] highlighted the overreliance on idealized channel models, reducing real-world applicability. Previous research Ref. [43] introduced a robust 3D clustering framework considering residual energy and spatial uncertainty, but without real sensor validation. Similarly, ref. [44] proposed IMP-RES-EL and EEL protocols, which improved lifetime by 36% and (cluster head) CH-to-BS (Base Station) transmission by 44% but lacked environmental integration. In contrast, the proposed EEL-GA protocol is designed for 3D WSNs in forest environments. It leverages GA-based CH optimization and integrates environmental datasets, offering a scalable and adaptive solution that bridges the gaps in existing LEACH variants.

### 2.1. Energy Efficiency vs. Energy Harvesting in WSNs

The energy efficiency in a WSN is increasingly prioritized over energy harvesting due to its broader, more consistent benefits and practical applicability. Although energy harvesting (EH) can supplement power in sensor nodes by collecting ambient sources, such as solar, wind, or vibration, it faces several technical and environmental limitations that restrict its reliability and scalability in complex deployments [45,46]. Additional EH sources include thermal gradients, piezoelectric effects, and radio frequency (RF) energy, each with unique constraints related to conversion efficiency, hardware integration, and ambient availability [47,48,49]. These limitations become more pronounced in obstructed, indoor, or remote natural environments where ambient energy is intermittent or insufficient.

Energy efficiency strategies, such as clustering-based routing, adaptive duty cycling, and load balancing, offer predictable and scalable solutions that directly reduce energy consumption without depending on variable ambient energy. Different studies show that energy-efficient methods reduce overall carbon emissions by lowering power requirements from non-renewable sources, contributing significantly to sustainability goals [50,51]. Furthermore, energy-efficient systems require lower infrastructure overhead than EH-enabled architectures, which often need Maximum Power Point Tracking (MPPT) and energy storage mechanisms [52]. From an economic standpoint, energy-efficient networks also prove more cost-effective. Efficient technologies reduce both installation and operational costs, offer quicker returns on investment, and require fewer maintenance interventions compared to EH systems that rely on external hardware and unpredictable environmental conditions [53]. Additionally, energy efficiency supports the creation of green jobs and promotes long-term economic sustainability by enabling energy-literate behaviors and policies [54].

Socially, energy-efficient systems improve indoor and ambient air quality, particularly in environments where inefficient energy use contributes to pollution. This leads to reduced health risks and better public well-being [55]. In contrast, EH techniques provide limited support for such social impacts, especially in low-light or obstructed settings where ambient energy capture is insufficient [48]. While energy harvesting remains a valuable complementary approach, especially in isolated or renewable-rich environments, the inherent uncertainties in energy supply and the hardware complexity involved make it less viable as a standalone solution for sustainable WSN operation. Therefore, this work focuses on optimizing energy efficiency through clustering and load balancing, which provides a more stable and scalable alternative to maintain WSN performance in real-world conditions. Table 1 highlights a direct comparison between energy efficiency and energy harvesting in WSNs, reinforcing the rationale behind the proposed energy-efficient model in this study.

In contrast to prior work, the current study proposes an EEL-GA protocol tailored specifically for 3D WSNs in forest environments, incorporating real-time datasets and optimizing for both energy and spatial metrics. Unlike existing LEACH-based hybrids that operate on ideal assumptions, our model dynamically responds to topological variation and environmental impact factors, thereby filling a critical research gap.

Table 2 summarizes key deployment-focused optimization methods used in WSN research.

### 2.2. Comparative Summary of Optimization Techniques

Table 3 highlights recent optimization-based CH selection protocols, including Firefly, Game Theory, and GA-based techniques. The proposed EEL-GA algorithm exhibits superior results in residual energy, transmission delay, and cluster longevity in 3D settings.

Recent studies have increasingly focused on nature-inspired optimization algorithms, such as Particle Swarm Optimization (PSO), the Genetic Algorithm (GA), Differential Evolution (DE), and the Artificial Bee Colony (ABC), due to their proven performance and popularity across a range of WSN applications. These algorithms have demonstrated high effectiveness in energy-efficient clustering and network lifetime improvement in wireless sensor networks [63]. For instance, DE and PSO are particularly noted for their superior performance in solving numerical optimization problems [64]. Each algorithm offers adaptability to complex optimization challenges: PSO mimics social behavior and dynamically explores the search space to optimize energy consumption strategies [65], while the GA is suitable for combinatorial tasks, such as cluster head selection. In terms of search mechanisms, GA uses crossover and mutation operations, DE employs differential mutations for faster convergence [66], and the ABC leverages foraging behavior for efficient local search and global exploration. These features also make them applicable in 3D forest deployments, where terrain and spatial irregularities affect sensor placement and performance [67]. Despite the availability of other metaheuristic techniques, a direct and comparative analysis of these four popular algorithms remains underexplored, which justifies their selection in the current study [63].

**Table 3:** Comparative summary of recent WSN optimization techniques.

Algorithm	Year	Key Features	Advantages
FCFAD [68]	2024	Fractal Clustering + Firefly Optimization	Enhances area coverage and connectivity
MFG-LEACH [69]	2024	Game-theoretic CH Selection	Reduces energy use; boosts packet delivery
IMP-RES-EL [70]	2024	Residual Energy-based CH Selection	Prolongs network life by up to 52%
ECH [39]	2024	Adaptive Duty Cycling	Reduces data redundancy; saves energy
AVOACS [71]	2024	African Vulture Optimization	Ensures stable, energy-balanced CH roles
3D-DEEC [72]	2024	Clustering for 3D UWSNs	Energy fairness in volumetric networks
SCHSM [73]	2024	Stochastic CH Selection	Maintains energy balance for IoT
EEL-GA (Proposed)	2025	GA-based CH Selection for 3D WSNs	Better energy retention, lifetime, and delay minimization

### 2.3. Deployment Challenges in 3D Forest Environments

WSNs deployed in forest terrains face unique challenges, notably due to obstacles like foliage, terrain elevation, and node inaccessibility. As discussed in [74], heterogeneous topologies, including relay and sink nodes, offer more resilient communication pathways. However, while 2D deployments have been well studied [75], 3D topologies require more sophisticated spatial modeling and clustering.

Random sensor placement is suitable for large, inaccessible areas but often leads to energy inefficiency. Predetermined deployment ensures controlled coverage but lacks scalability. Thus, optimization-based deployments tailored for 3D terrains are essential.

### 2.4. Recent Advances in Sensor Deployment for 3D Environments

Recent studies employ bio-inspired algorithms for 3D WSN optimization. Works such as [76,77] explore coverage-efficient deployment via evolutionary models. Others, like [78,79], propose polynomial-time heuristics for constrained 3D sensor grids.

PSO remains a favored approach for sensor positioning and adaptive clustering in 3D environments. Studies [80,81] highlight its adaptability in mobile and underwater WSNs. Similarly, ABC algorithms have also been explored for dynamic CH selection and energy-efficient node management.

The literature consistently supports the need for hybrid optimization strategies to address the multi-constraint nature of 3D deployments. Our proposed EEL-GA aligns with these trends and extends them through real-world environmental data integration and a fitness-based, multi-metric clustering approach.

## 3. Structure of Wireless Sensor Node

**Figure 1:**
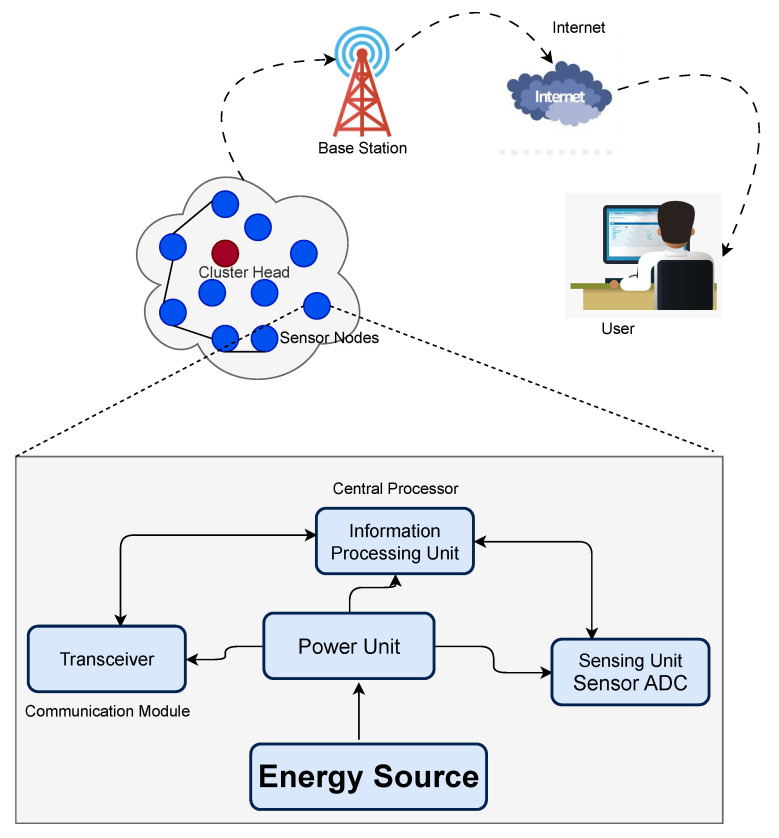
Internal components of a typical wireless sensor node.

In a WSN, the architecture of a sensor node is central to enabling efficient data acquisition, processing, and transmission. As shown in Figure 1, each sensor node is powered by an energy source that supplies power to its internal components. The energy is distributed to key subsystems, including the Sensing Unit, Central Processor (Information Processing Unit), Power Unit, and the Communication Module (Transceiver). These integrated components enable the node to perform sensing, processing, and wireless communication, supporting real-time environmental monitoring and efficient participation in cluster-based communication protocols.

Sensor nodes capture data from the surrounding environment, perform in-node processing to reduce redundancy, and transmit the information to the BS (Base Station), either directly or via multi-hop clustering protocols, such as LEACH. The Base Station (BS) interfaces with the user through the Internet, allowing remote access to sensor data. This integrated architecture ensures energy efficiency, adaptability, and scalability for deployment in complex environments.

### 3.1. Node Roles in Hierarchical WSNs

A hierarchical WSN architecture typically comprises four distinct node roles. Regular sensor nodes are responsible for sensing environmental parameters and transmitting the data to a designated CH; they are energy-constrained and constitute the bulk of the network [82]. CHs handle the aggregation and compression of data received from their member nodes and forward it to the Base Station; due to their critical role, CHs experience higher energy consumption [83]. In multi-hop communication scenarios, some CHs may function as CH-to-sink forwarders, relaying data from more remote CHs toward the BS. Finally, the BS serves as the central data sink, typically positioned either inside or outside the monitored area, and is equipped with unlimited energy and computational resources [84].

### 3.2. Communication Types in WSNs

The communication architecture in hierarchical WSNs typically involves three primary patterns. Intra-cluster communication refers to the process where member nodes transmit their sensed data to the respective CHs using short-range links, thereby conserving energy. Inter-cluster communication occurs when CHs relay data to adjacent CHs, which is particularly beneficial in large-scale or obstructed terrains that necessitate multi-hop transmissions. Finally, CH-to-sink communication involves the transmission of aggregated data from CHs to the BS, which may be performed through either single-hop or multi-hop strategies depending on the network’s routing protocol [85].

### 3.3. 3D Sensor Deployment Strategy

The deployment strategy incorporates both random and fitness-based spatial techniques. Initially, sensor nodes are randomly distributed within a $1\;{\mathrm{km}}^{3}$ three-dimensional cubic space, simulating unpredictable aerial deployment scenarios [86]. Following this, a fitness-based spatial evaluation is applied where node positions are assessed based on metrics such as residual energy and intra-cluster distances [87]. This approach ensures that nodes with higher energy levels are positioned at optimal locations to serve as CHs, thereby enhancing overall network efficiency and lifetime.

### 3.4. Energy-Aware Operation Framework

Efficient energy management extends the operational lifetime of the WSN. The proposed system integrates several strategies. The LEACH (Low-Energy Adaptive Clustering Hierarchy) protocol facilitates dynamic role rotation among sensor nodes, effectively balancing energy consumption throughout the network [15]. CHs employ data aggregation techniques, such as fusion algorithms, to eliminate redundant transmissions and optimize bandwidth usage [88]. Additionally, sleep scheduling mechanisms allow idle nodes to switch to low-power modes based on their activity cycles, significantly reducing unnecessary energy consumption [89]. In more advanced WSNs, mobility support can be integrated through mobilizers that reposition nodes in response to environmental variations or node failures, enhancing network adaptability.

This structured, layered design, coupled with dynamic clustering and energy-centric deployment, renders the WSN resilient, sustainable, and suitable for smart forestry and other real-time monitoring applications in challenging 3D environments.

## 4. Design Model

**Figure 2:**
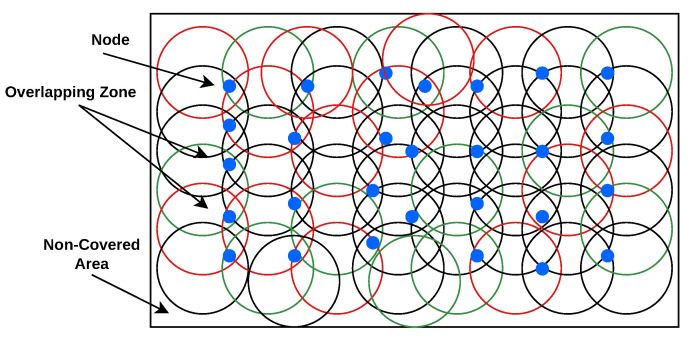
Conceptual 2D projection of sensor node deployment illustrating sensing ranges, overlapping coverage zones, and gaps. This view simplifies spatial understanding before transitioning to 3D deployment.

**Figure 3:**
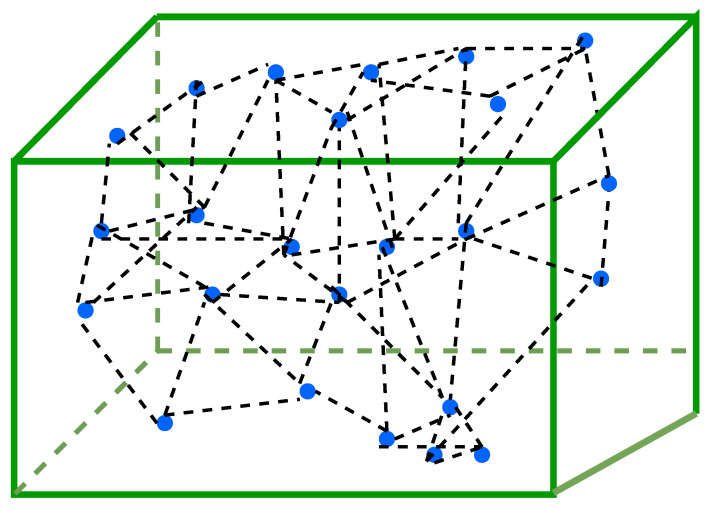
3D sensor deployment in forested terrain.

For clarity of visualization, Figure 2 first illustrates a 2D projection of sensor node deployment, showing overlapping coverage zones, clustering, and non-covered regions. This simplified view helps highlight the limitations of earlier 2D models used in previous studies. Building on this, the present work considers the challenge of deploying wireless sensor nodes in a realistic 3D forest environment, as illustrated in Figure 3. A total of *N* sensor nodes (e.g., environmental or image sensors) are deployed to ensure volumetric coverage. These nodes are responsible for sensing environmental parameters and forwarding the collected data to the BS through relay nodes. Relay nodes serve as intermediate communication hubs, helping balance the energy burden across the network and extending the operational lifetime of the WSN.

### Deployment Position of Sensors

Sensor nodes are strategically placed on trees or elevated forest structures to optimize visibility and connectivity in a three-dimensional space. For planning and visualization, the 3D coordinates of deployed nodes are mapped onto a 2D plane.

This 2D projection serves as a conceptual aid to illustrate sensing coverage and overlap in a simplified view, offering an abstract representation of spatial deployment prior to introducing the full 3D environment. As shown in (Figure 2), sensor nodes (blue circles) are distributed across the area, each with a sensing range represented by a red, green, or black circle. Overlapping zones indicate regions where multiple nodes provide coverage, while non-covered areas represent sensing gaps caused by irregular deployment.

$$\begin{array}{cc} b_{x} & =l-1,\;b_{y}=w-1,\;b_{z}=h-1 \end{array}$$

Here, l,w,h denote the length, width, and height of the forested area, respectively. We consider a 3D deployment space defined by the bounds (x,y,z)∈[0,l−1]×[0,w−1]×[0,h−1]. Sensor nodes are probabilistically deployed by sampling 3D coordinates using a fitness-guided probability function. This approach does not rely on a fixed 2D-to-3D mapping, but instead uses energy and spatial criteria to bias node placement for efficient clustering.

(1) $$P\left(x,y,z\right)=\frac{\sigma_{\mathrm{energy}}\left(x,y,z\right)+D_{\mathrm{intra}}\left(x,y,z\right)}{\sum_{k=1}^{N}\left(\sigma_{\mathrm{energy}}\left(x_{k},y_{k},z_{k}\right)+D_{\mathrm{intra}}\left(x_{k},y_{k},z_{k}\right)\right)}$$

Here, $\sigma_{\mathrm{energy}}$ represents the normalized residual energy of a node, $D_{\mathrm{intra}}$ denotes the intra-cluster distance to candidate CHs, and *N* is the total number of nodes in the network. This probabilistic distribution promotes the selection of nodes with higher residual energy and better cluster centrality, supporting efficient cluster formation and enhanced communication longevity.

Figure 2 illustrates a 2D top-view projection (*x*–*y* plane) of the randomly deployed sensor nodes, primarily for visualization clarity. This is followed by the full 3D layout shown in Figure 3, where each node is extended along the *z*-axis to reflect elevation differences in the forest environment. The 2D projection helps identify node clustering and spatial distribution from above, while the 3D layout provides a complete view of deployment in a realistic terrain.

## 5. Sensing Model Approach

To accurately model coverage in a 3D setting, the sensing model considers both the Euclidean distance and directional angles. The probability of a sensor *r* detecting a point *q* is defined as

(2) $$P\left(r,q\right)=P_{\alpha }\left(r,q\right)\cdot P_{\beta }\left(r,q\right)\cdot P_{\gamma }\left(r,q\right).$$

Here, $P_{\alpha }\left(r,q\right)$ represents the sensing probability based on radial distance, $P_{\beta }\left(r,q\right)$ accounts for the horizontal angular position, and $P_{\gamma }\left(r,q\right)$ captures vertical angular alignment. These three components together determine the overall sensing probability in a 3D spatial environment.

This separable angular model (adapted from [90]) enables more realistic 3D volume coverage, especially in dense forest environments. Unlike traditional elliptical sensing approximations, this model assumes a rectangular field-of-view, offering a more accurate representation of sensor coverage in complex terrains.

### 5.1. Network Assumption

The WSN operates within a 1 km^3^ forested region, where *N* homogeneous and stationary nodes are randomly distributed. Each node is aware of its location and initial energy. The BS is located at a fixed coordinate, typically at the corner or center of the region.

Node positions are sampled using

$$x_{i}∼U\left(0,L\right),\;y_{i}∼U\left(0,W\right),\;z_{i}∼U\left(0,H\right).$$

Clustering is performed periodically using the proposed EEL-GA approach, based on two principal criteria:

1. Residual energy of nodes;
2. Intra-cluster distance to the CH.

According to [91] the fitness function for cluster head selection is

$$F_{i}=w_{1}\cdot \frac{E_{i}\left(r\right)}{\bar{E}\left(r\right)}+w_{2}\cdot \left(1-\frac{d_{i}}{d_{max}}\right).$$

In this context, $E_{i}\left(r\right)$ represents the residual energy of node *i* at round *r*, while $\bar{E}\left(r\right)$ denotes the average energy of the entire network during the same round. The term $d_{i}$ refers to the average distance between node *i* and its associated cluster members, and $d_{max}$ defines the maximum allowable intra-cluster distance threshold for maintaining efficient communication. Nodes with the highest fitness scores are selected as CHs, with reclustering triggered when energy levels or distances exceed predefined limits.

### 5.2. Energy Consumption Model for Data Transmission and Reception

This study adopts the first-order radio model [15] to evaluate energy usage for both transmission and reception.

(3) $$E_{Tx}\left(k,d\right)=E_{elec}\cdot k+E_{amp}\cdot k\cdot d^{2},\;E_{Rx}\left(k\right)=E_{elec}\cdot k$$

Here, *k* represents the packet size in bits, and *d* denotes the communication distance. The parameter $E_{elec}$ refers to the energy consumed by the electronic circuitry for processing each bit, while $E_{amp}$ signifies the energy required for signal amplification over the transmission distance.

**Figure 4:**
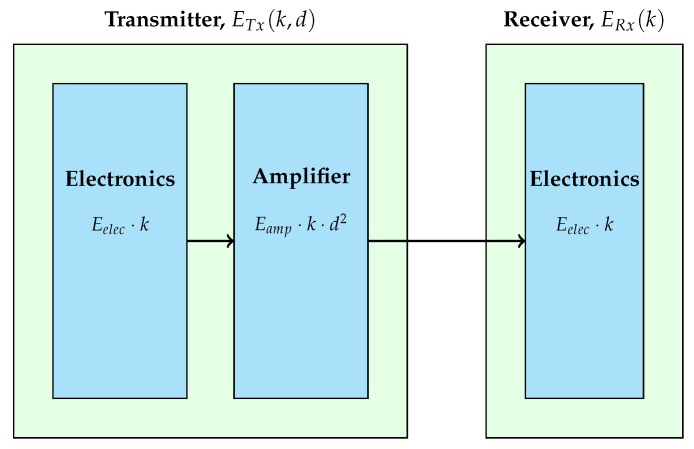
Radio energy dissipation model showing energy cost for transmission and reception in WSN nodes.

The radio energy dissipation model, which shows the energy cost for transmission and reception in WSN nodes, is illustrated in Figure 4. This energy model is integrated into the clustering decision-making, influencing CH selection by penalizing nodes with low energy or long-range communication burdens. In this model, sensor nodes are assumed to be randomly deployed across the 3D forested region using a uniform distribution. Although placement is stochastic, each node remains stationary after deployment, reflecting realistic forest monitoring setups where sensors are typically mounted on trees or fixed platforms. Furthermore, each node is assumed to be aware of its position (e.g., via GPS or anchor-based localization) to facilitate accurate distance calculations during clustering. This combination of random deployment and position awareness ensures realistic spatial modeling while supporting energy-aware routing and cluster formation mechanisms in the proposed EEL-GA framework.

### 5.3. Sensing Distance Modeling

The sensing probability due to distance is modeled as [92]

(4) $$P_{\alpha }\left(r,q\right)=\left\{\begin{array}{cc} 1, & \delta \in [0,R_{\delta }]\\ \exp\left(-{\left(\frac{\delta -R_{\delta }}{R_{\max}-R_{\delta }}\right)}^{\mu /\delta }\right)\\ \times \left(1+\tan{\left(\frac{\delta -R_{\delta }}{R_{\max}-R_{\delta }}\right)}^{\nu /\delta }\right), & \delta \in (R_{\delta },R_{\max}]\\ 0, & \delta >R_{\max} \end{array}\right..$$

Here [77], δ is the Euclidean distance between the sensor and target:

$$\delta =\sqrt{{\left(x_{r}-x_{q}\right)}^{2}+{\left(y_{r}-y_{q}\right)}^{2}+{\left(z_{r}-z_{q}\right)}^{2}}.$$

This model reflects the gradual decline in sensing efficiency with increasing distance and aligns well with forested propagation environments.

### 5.4. Cluster Head Selection Using LEACH

According to [15] LEACH selects CHs probabilistically using the threshold by

$$T\left(n\right)=\left\{\begin{array}{cc} \frac{p}{1-p\cdot (r\;\mathrm{mod}\;\frac{1}{p})}, & n\in G\\ 0, & \mathrm{otherwise} \end{array}\right.,$$

where

- *p*: desired CH probability;
- *r*: current round;
- *G*: nodes not selected as CHs in the last 1/p rounds.

This ensures fair role rotation among nodes. In our EEL-GA hybrid model, this baseline LEACH strategy is enhanced using a GA-based fitness function to select more optimal and energy-efficient CHs across the 3D space.

## 6. Energy-Efficient LEACH Genetic Algorithm (EEL-GA)

This section presents the proposed hybrid clustering approach, EEL-GA, which combines the LEACH protocol with GA optimization to enhance energy efficiency in 3D WSNs. The goal is to intelligently select CHs that are energy-rich, spatially central, and load-balanced, thereby extending the network lifetime.

### 6.1. Unified Dual-Metric Fitness Function for CH Selection

In the proposed Genetic Algorithm, each chromosome represents a set of candidate CHs. A multi-objective fitness function evaluates the quality of each solution by considering three key factors: residual energy, distance to the BS, and cluster load [93]:

(5) $${\mathrm{Fitness}}_{i}=w_{1}\cdot \frac{E_{i}}{E_{\max}}-w_{2}\cdot \frac{D_{i,\mathrm{BS}}}{D_{\max}}-w_{3}\cdot \frac{1}{N_{i}}.$$

Here, $E_{i}$ is the residual energy of CH *i*, $D_{i,\mathrm{BS}}$ is the distance from CH *i* to the BS, and $N_{i}$ is the number of member nodes in the cluster. The weights $w_{1},w_{2},w_{3}$ are positive and satisfy $w_{1}+w_{2}+w_{3}=1$, determining the influence of each metric. This initial fitness evaluation Equation (5) is used to preselect candidate CHs before evolving them using the GA-based fitness function in Equation (6).

The first term promotes energy-efficient CHs, the second minimizes communication cost, and the third helps balance cluster loads. This combination ensures the selection of CHs that extend network lifetime, reduce energy consumption, and prevent node overloading [94].

To select suitable values for the weights, multiple combinations were tested through simulation. Each set of weights was evaluated based on its impact on energy efficiency, network lifetime, and load balancing. The final combination was chosen because it showed the best overall performance across these three aspects. While more advanced methods, such as Pareto optimization, could be applied in future work, this study focused on practical testing to determine effective values.

### 6.2. Flowchart of EEL-GA Execution

**Figure 5:**
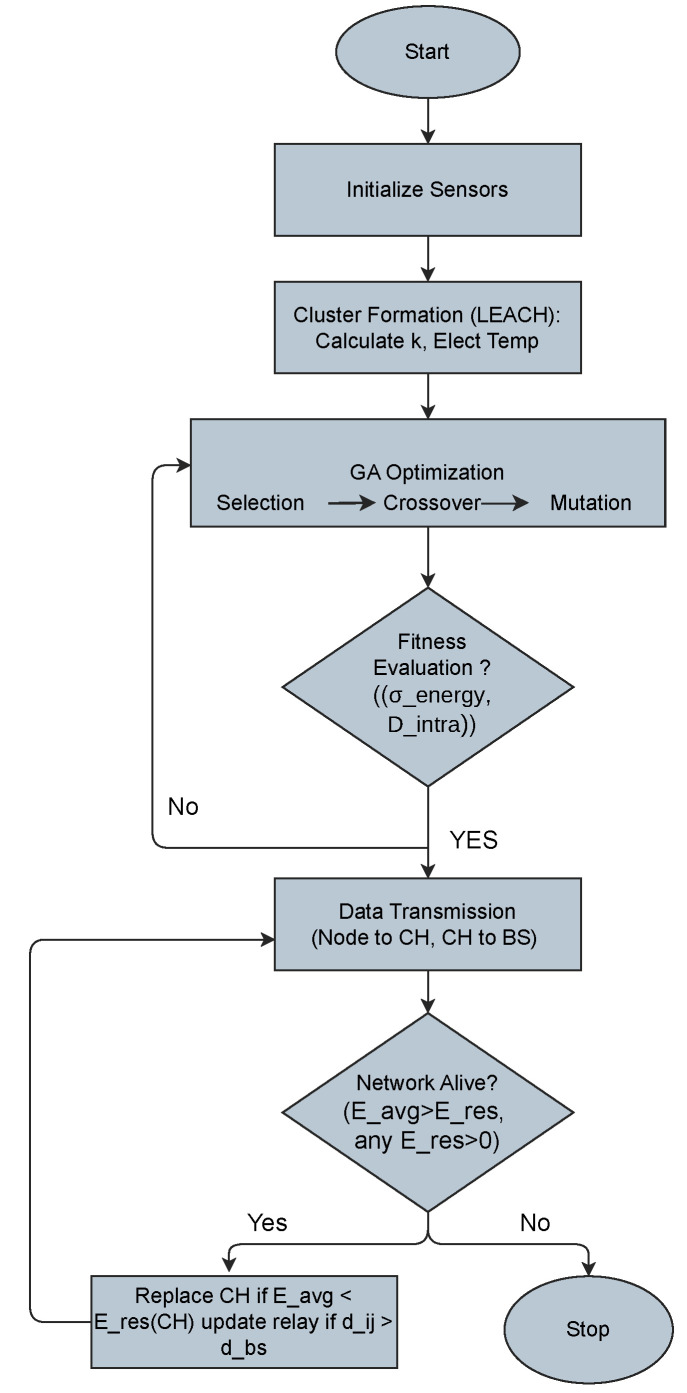
Logical flow of the proposed EEL-GA algorithm showing LEACH-based clustering, genetic optimization, and reclustering triggered based on CH energy and communication distance.

To illustrate the logical flow of the EEL-GA algorithm in 3D WSN, Figure 5 shows the hybrid procedure designed to optimize energy-efficient communication. The process starts with node deployment and cluster formation using LEACH. The GA refines cluster head (CH) selection by evaluating energy and distance-based fitness metrics. This fitness evaluation is performed using the dual-metric function described in Equation (6), while Equation (5) was initially explored for multi-objective evaluation, which balances residual energy fairness and spatial compactness for optimal CH selection. If CHs are not optimal, the process iterates using genetic operations (selection, crossover, mutation). Once CHs are selected, data transmission takes place from nodes to CHs and from CHs to the BS. The system periodically checks network vitality and triggers reclustering if any CH has energy below average or exceeds distance thresholds. The loop continues until the network is no longer operational.

### 6.3. Algorithm Description

The EEL-GA operational framework is summarized in Algorithm 1. It iteratively updates the network topology through reclustering and energy monitoring. Each round evaluates and evolves CH configurations based on fitness scores derived from node characteristics and environmental conditions.
**Algorithm 1** EEL-GA: Energy-Efficient LEACH Clustering using the Genetic Algorithm.**Input:** Number of nodes *N*, initial energy $E_{0}$, population size *P*, number of generations *G*, cluster probability *p*, number of clustering rounds *R*, 3D area parameters, environmental dataset
**Output:** Optimized cluster head list CH, energy metrics, performance statistics
  1:Deploy *N* sensor nodes randomly within a 1000 m × 1000 m × 1000 m region  2:Initialize energy and load environment parameters (e.g., temperature, humidity)  3:Perform initial CH evaluation using Equation (5) based on residual energy, distance to BS, and cluster load  4:Generate initial GA population representing CH configurations of size *P*  5:Set generation count g=1  6:**for** each round r≤R **do**  7:    Evaluate node failures based on energy threshold  8:    Calculate $\sigma_{\mathrm{energy}}$ and $D_{\mathrm{intra}}$ for fitness evaluation  9:    Adjust environmental impact using dataset parameters10:    **while** g≤G **do**11:        Evaluate population using$$f=\alpha \cdot \sigma_{\mathrm{energy}}+\left(1-\alpha \right)\cdot D_{\mathrm{intra}}$$12:        Select parents based on fitness13:        Apply crossover and mutation to generate offspring14:        Replace worst individuals with offspring15:        g=g+116:    **end while**17:    Select best individual (CH configuration)18:    Form clusters by assigning nodes to nearest CH19:    Calculate energy consumption using$$E_{Tx}\left(k,d\right)=E_{elec}\cdot k+E_{amp}\cdot k\cdot d^{2}$$20:    Update node energy, collect QoS metrics21:**end for**22:**return** Final CH set, clustering results, and performance indicators


### 6.4. Evolution Strategy and Dual-Metric Fitness Function

To ensure spatial compactness and balanced energy distribution among CHs, a dual-metric fitness function is employed:

(6) $$F=\alpha \cdot \sigma_{\mathrm{energy}}+\left(1-\alpha \right)\cdot D_{\mathrm{intra}}.$$

In this fitness function, the weighting coefficient α plays a crucial role in balancing energy fairness ($\sigma_{\mathrm{energy}}$) and spatial compactness ($D_{\mathrm{intra}}$) during cluster head (CH) selection. To determine an appropriate value for α, we conducted iterative empirical testing over a range from 0.1 to 0.9 (in increments of 0.1). Each setting was evaluated based on its impact on residual energy, cluster lifetime, and packet delivery ratio. Among these, α=0.6 provided the best overall trade-off between energy conservation and spatial efficiency in the 3D forest deployment and was therefore adopted for the final implementation.

The parameters used in the fitness evaluation include the following: $\sigma_{\mathrm{energy}}$, which represents the standard deviation of residual energies among the CHs, reflecting energy fairness; $D_{\mathrm{intra}}$, denoting the average intra-cluster distance that influences spatial compactness; and α, a weighting factor that balances the trade-off between energy distribution and spatial efficiency.

Each generation applies

(7) $$C_{\mathrm{new}}=\mathrm{Crossover}\left(C_{1},C_{2}\right),\;C_{\mathrm{mutated}}=\mathrm{Mutation}\left(C_{\mathrm{new}}\right).$$

Diversity in the population is maintained via mutation, while elite chromosomes guide convergence [95].

### 6.5. Clustering and Spatial Communication

**Figure 6:**
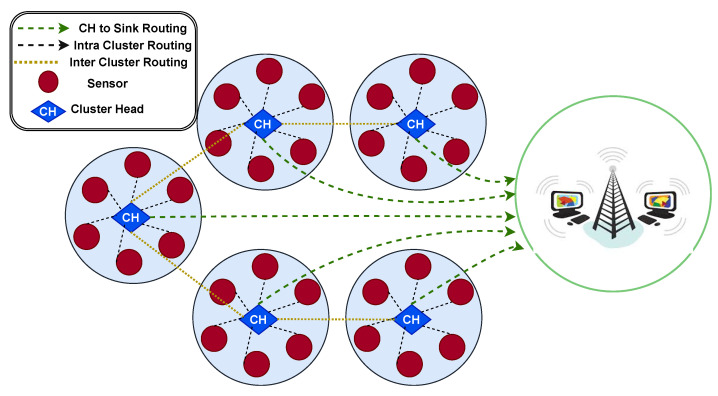
Clustered WSN communication topology under EEL-GA: sensor nodes (red circles), cluster heads (blue diamonds), intra-cluster communication (black dashed lines), inter-cluster communication (yellow dashed lines), and CH-to-sink routing (green dashed lines).

The proposed EEL-GA framework organizes the wireless sensor network into multiple clusters. Each cluster comprises several sensor nodes (depicted as red circles) and one designated cluster head (CH), represented by a blue diamond. As shown in Figure 6, sensor nodes transmit their collected data to their respective CHs through intra-cluster communication (black dashed lines). After data aggregation, CHs forward the information either directly to the BS using CH-to-sink communication (green dashed lines) or via neighboring CHs through inter-cluster communication paths (yellow dashed lines), depending on routing conditions and energy considerations. These communication paths are optimized by the proposed EEL-GA algorithm. While the framework builds upon the traditional LEACH architecture, it replaces random CH selection and static single-hop transmission with a fitness-based Genetic Algorithm that considers residual energy, spatial positioning, and dynamic environmental variables [96]. This approach enables energy-efficient, adaptive routing across complex 3D forest deployments.

This structure not only improves energy efficiency but also provides robustness against node failures. If a sensor node or CH fails, neighboring clusters can reconfigure or redirect traffic adaptively, maintaining communication continuity in the network.

### 6.6. Fitness-Based CH Selection with Spatial Metrics

In complex terrains, CHs are selected based on a composite metric that combines residual energy, node density, distance to the BS, and link quality—ensuring robust, energy-efficient deployment under environmental heterogeneity [97]:

(8) $$f=\Phi_{1}z_{1}+\Phi_{2}z_{2}+\Phi_{3}z_{3},\;\Phi_{3}=1-\Phi_{1}-\Phi_{2}.$$

The composite fitness metric incorporates three essential clustering objectives. The first component, $z_{1}$, represents the average 3D distance to the cluster head, promoting spatial compactness and reducing intra-cluster communication cost. The second component, $z_{2}$, captures energy balance within the cluster, computed as $\frac{E_{\mathrm{avg}}C_{n}}{E_{p}}$, and encourages the selection of nodes with higher residual energy, thereby extending the network’s lifetime. The third component, $z_{3}$, defined as the inverse of the cluster size $\left(\frac{1}{C_{n}}\right)$, helps regulate the cluster load by penalizing disproportionately large clusters. Together, these components ensure energy-aware, balanced, and spatially efficient clustering.

### 6.7. Genetic Population Evolution Strategy

The EEL-GA protocol applies classical Genetic Algorithm operations to evolve a population of candidate CH configurations across generations. Each chromosome encodes a potential set of CHs. During each generation, the following takes place:

- Selection: The fittest chromosomes (based on residual energy and intra-cluster distance) are selected using tournament or roulette-based methods.
- Crossover: Selected parents exchange segments to create offspring, encouraging exploration of new CH combinations.
- Mutation: With a small probability, gene values are randomly altered to maintain diversity and prevent premature convergence.
- Replacement: Weaker chromosomes are replaced by fitter offspring, ensuring that the population progressively evolves toward optimal clustering configurations.

This evolutionary process continues over fixed generations and yields a final CH set with balanced energy usage and optimized spatial layout.

### 6.8. Simulation Setup

The simulation environment was implemented in MATLAB R2024b and configured based on the parameters listed in Table 4. The 3D deployment was randomized across a simulated forest terrain of 1000 m × 1000 m × 1000 m to reflect vertical and horizontal irregularities commonly found in real forest ecosystems. Node energy levels were initialized using a normal distribution around 0.5 J to model non-uniform deployment states. To ground our model in real-world constraints, the environmental conditions were derived from an actual deployment in the Juniper Forest of Ziarat, Balochistan. The recorded parameters, including temperature, humidity, soil moisture, wind speed, and light, were used to adjust signal propagation factors, energy consumption behavior, and packet loss simulations. For example, higher humidity levels degraded the radio signal strength, influencing the path loss exponent dynamically within the simulation. This tuning allowed us to evaluate the robustness and adaptability of the EEL-GA protocol under forest-like environmental stressors.

As shown in Table 4, a small population size of 10 is selected to reduce computational complexity and energy usage during cluster setup. This choice is suitable for WSN scenarios, where faster convergence and lower overhead are critical. Previous studies [98,99] have also demonstrated that small population sizes can be effective in achieving optimization results achieving best-known fitness within 10 generations, as validated in prior WSN studies using small population sizes, i.e., 4–14 as shown in [100]. The main simulation parameters and their selection rationale are summarized in Table 5.

### 6.9. Environmental Impact on WSN Operations and EEL-GA Adaptations

Environmental conditions significantly influence the performance and reliability of WSNs. Key factors such as temperature, humidity, and precipitation can degrade communication quality and reduce node longevity. The EEL-GA algorithm incorporates adaptive strategies to mitigate these effects, as summarized in Table 6.

Studies have shown that higher temperatures can reduce wireless communication quality, requiring increased transmission power to maintain stable connections [101]. Similarly, high humidity levels can cause signal attenuation, leading to increased packet loss and retransmissions. Precipitation, such as rainfall, can physically damage sensor nodes and further degrade signal propagation [102]. To address these challenges, EEL-GA dynamically adjusts node operations, including duty cycling, transmission power, and routing decisions, based on real-time environmental data, thereby enhancing network resilience and longevity.

## 7. Results and Discussion

This section provides a comprehensive performance evaluation of the proposed Energy-Efficient LEACH with the Genetic Algorithm (EEL-GA) protocol in comparison with three other LEACH-based hybrid optimization methods: EEL-PSO (Particle Swarm Optimization), EEL-DE (Differential Evolution), and EEL-ABC (Artificial Bee Colony). The analysis is based on key metrics including residual energy, cluster lifetime, scheduling overhead, average transmission delay, and the packet delivery rate. Simulations were conducted using MATLAB with realistic node deployment scenarios derived from the real-time dataset.

### 7.1. Scheduling Overhead vs. Node Density

**Figure 7:**
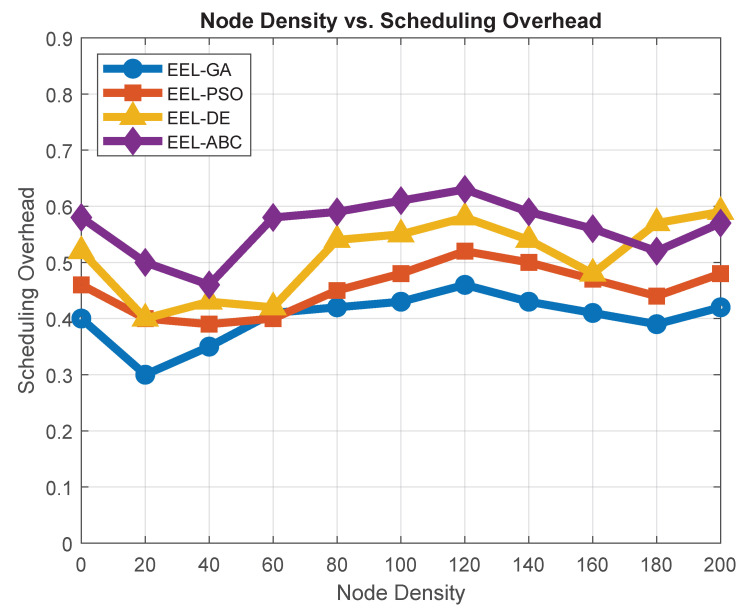
Scheduling overhead vs. node density.

Scheduling overhead refers to the number of control packets exchanged during cluster formation and maintenance, such as cluster head (CH) selection notifications, cluster join requests, and periodic status updates. In this study, the overhead is measured by counting the cumulative number of these control messages generated in each round, excluding standard data transmissions. As shown in Figure 7, the EEL-GA protocol exhibits the lowest scheduling overhead across all tested node densities, outperforming EEL-PSO, EEL-DE, and EEL-ABC. This efficiency results from EEL-GA’s optimized clustering process, which reduces redundant control signaling by selecting stable and energy-efficient CHs. Minimizing scheduling overhead is critical in WSNs, particularly in forest environments, where excessive control communication can lead to unnecessary energy expenditure and increased contention on wireless channels.

### 7.2. Residual Energy vs. Node Density

**Figure 8:**
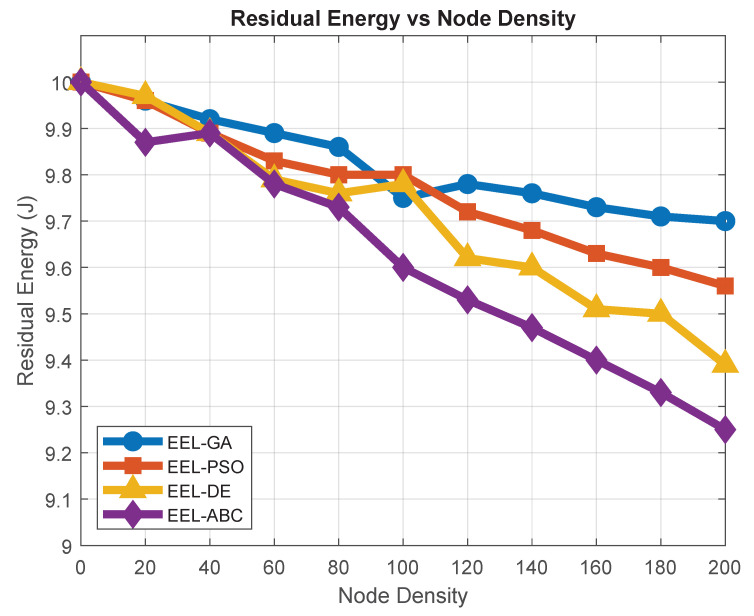
Residual energy vs. node density.

Residual energy is a direct measure of how efficiently a WSN conserves power during operation. In this study, residual energy is calculated by summing the remaining energy across all sensor nodes at the end of each simulation round. Higher residual energy values indicate lower overall energy consumption and better sustainability of the protocol. As depicted in Figure 8, the proposed EEL-GA approach consistently preserves more residual energy than the comparative clustering protocols: EEL-PSO, EEL-DE, and EEL-ABC. This performance can be attributed to the dual-objective fitness function employed in EEL-GA, which favors the selection of cluster heads (CHs) with higher remaining energy and closer proximity to their members. This energy-aware clustering strategy prevents overburdening weaker nodes, resulting in a more balanced energy expenditure throughout the network. The results clearly demonstrate EEL-GA’s effectiveness in extending the network lifetime, particularly under increased node density scenarios where energy efficiency becomes more critical. To further validate the significance of the observed differences, a one-way ANOVA followed by Tukey’s post hoc test was conducted on the residual energy values across all optimization methods. The results confirmed that the differences were statistically significant (*p* < 0.05), reinforcing the superiority of EEL-GA in maintaining higher residual energy under varying network densities.

### 7.3. Cluster Lifetime vs. Number of Clusters

**Figure 9:**
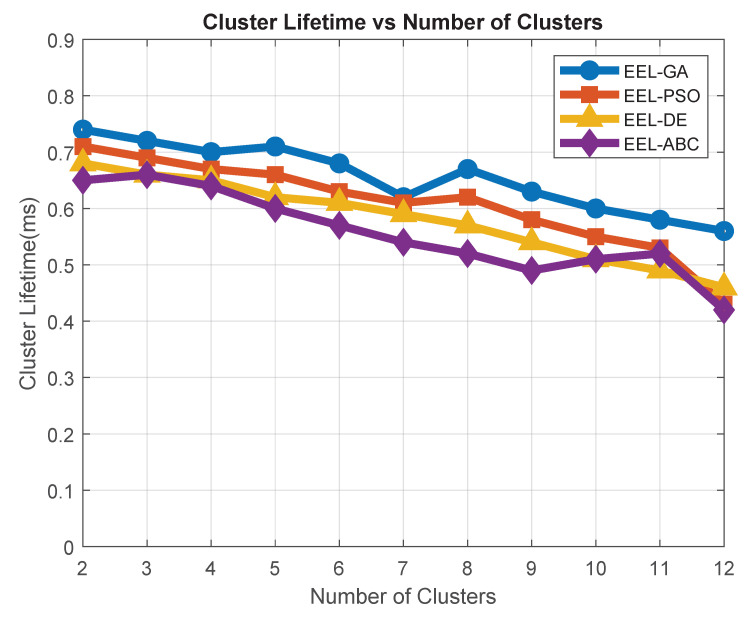
Cluster lifetime vs. number of clusters.

Cluster lifetime reflects the operational duration of a cluster before it becomes inactive due to energy depletion or node disconnection, whereas cluster lifetime is measured by tracking the number of simulation rounds during which a given cluster remains active. A cluster is considered active as long as its designated CH remains functional and maintains at least one member node. Once the CH fails or all members leave or die, the cluster is marked inactive, and the lifetime is recorded. As shown in Figure 9, the EEL-GA algorithm consistently maintains longer-lasting clusters compared to EEL-PSO, EEL-DE, and EEL-ABC. The *x*-axis represents the number of clusters formed in the network, while the *y*-axis indicates the average operational lifetime of these clusters measured across simulation rounds. This improvement is primarily due to EEL-GA’s CH selection mechanism, which takes into account both the residual energy and spatial proximity of nodes. By preventing the assignment of high-load roles to low-energy nodes and maintaining compact cluster formations, the protocol minimizes early CH failures. As a result, the network remains structurally stable for a longer period, supporting prolonged monitoring operations in energy-constrained forest environments.

### 7.4. Average Transmission Delay vs. Node Density

**Figure 10:**
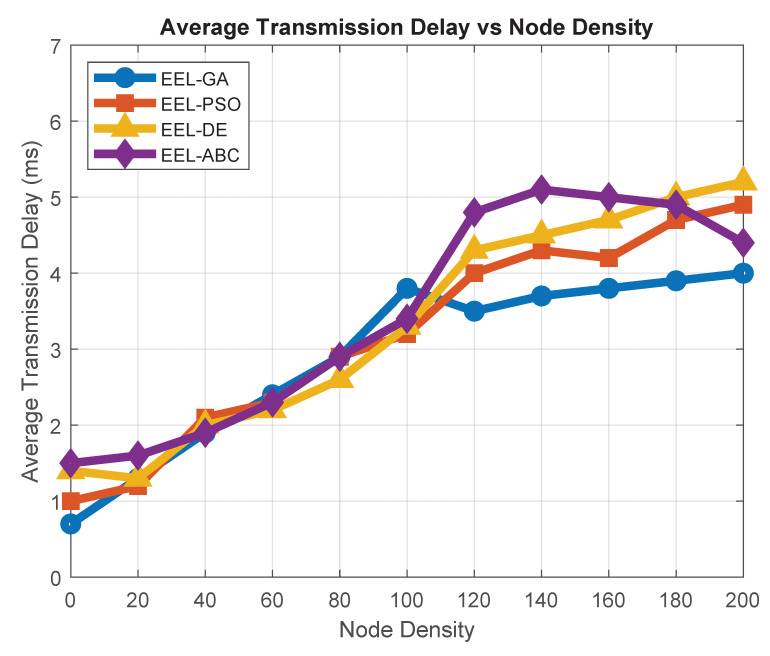
Average transmission delay vs. node density.

Average transmission delay quantifies the time required for data packets to reach the BS from sensor nodes. The average transmission delay is measured by recording the time each data packet is generated at the source node and the time it is successfully received at the BS. The delay for each packet is computed as the difference between these two timestamps, and the average is taken across all successfully delivered packets during the simulation. This measurement simply captures the time difference between when a data packet is generated at the source node and when it is successfully received at the BS, averaged across all delivered packets. Lower delay indicates more efficient routing and reduced communication latency. As depicted in Figure 10, EEL-GA consistently outperforms other algorithms by achieving minimal delays, particularly in scenarios with high node density. This improvement stems from EEL-GA’s ability to establish compact clusters and select CHs that are optimally positioned. Consequently, the protocol minimizes intra-cluster communication distances, enabling faster data transfer and supporting latency-sensitive WSN applications.

### 7.5. Packet Delivery Ratio vs. Node Density

**Figure 11:**
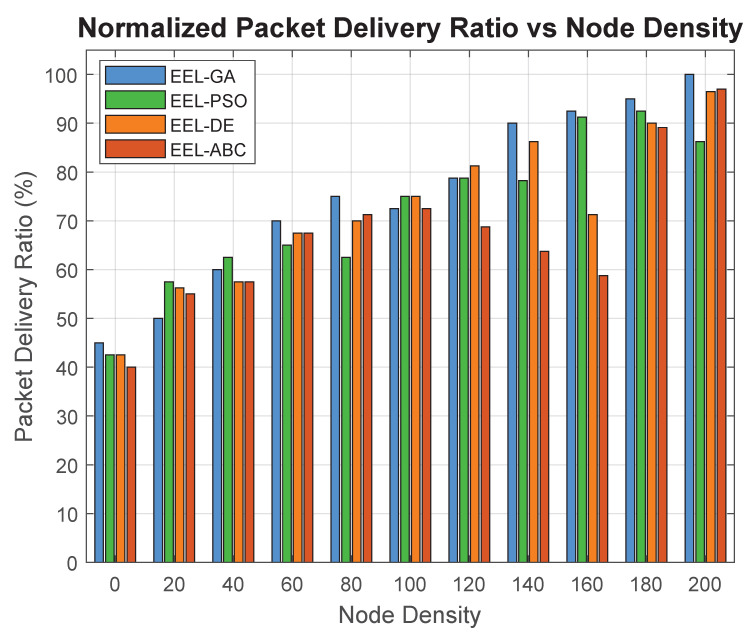
Packet delivery ratio vs. node density.

Figure 11 illustrates the relationship between the node density and packet delivery ratio (PDR) across four clustering protocols: EEL-GA, EEL-PSO, EEL-DE, and EEL-ABC. In this work, the packet delivery ratio (PDR) is defined as the amount of data (in kilobits) successfully delivered to the BS per second, measured in kilobits per second (kbps). It is calculated by measuring the total volume of correctly received packets at the BS over time and dividing it by the total simulation duration, providing a reliable indicator of throughput. The *x*-axis represents the number of active sensor nodes in the network, while the *y*-axis measures the PDR in kilobits per second (kbps). As node density increases, all algorithms exhibit a general improvement in PDR, indicating enhanced data throughput due to better network connectivity. However, EEL-GA consistently outperforms the other methods across all densities. This performance advantage stems from its intelligent CH selection strategy based on the residual energy and intra-cluster distance, which improves data aggregation and reduces packet loss. Compared to EEL-PSO, EEL-DE, and EEL-ABC, the EEL-GA protocol demonstrates more efficient routing and fewer dropped packets, particularly in high-density deployments. These results confirm its effectiveness for large-scale forest monitoring scenarios where high data reliability is essential.

## 8. Conclusions

This paper presented an enhanced clustering framework, EEL-GA, for energy-efficient communication in three-dimensional Wireless Sensor Networks (WSNs) deployed in forest environments. The proposed protocol integrates the classic LEACH model with a Genetic Algorithm to enable adaptive and energy-aware cluster head (CH) selection based on residual energy and intra-cluster distance metrics. Through simulation experiments grounded in real-world data collected from the Juniper Forest of Ziarat, Balochistan, the EEL-GA protocol demonstrated significant improvements in key performance indicators, including scheduling overhead, residual energy conservation, cluster lifetime, transmission delay, and packet delivery rate. Unlike traditional clustering models designed for flat or 2D surfaces, the EEL-GA approach accounts for the topological complexity of forest environments by simulating deployment over a 3D terrain. This not only improves the spatial accuracy of routing decisions but also ensures energy load balancing by preventing overuse of high-elevation or isolated nodes. By incorporating environmental influences, such as temperature and humidity, into the simulation parameters, the model closely mimics field conditions, improving its practical relevance and reliability. Adaptive mechanisms embedded within EEL-GA ensure robustness against signal degradation and dynamic node conditions—critical for applications like smart forestry where reliability and longevity are essential. The comparative analysis further validated EEL-GA’s superiority over similar hybrid optimization techniques, such as EEL-PSO, EEL-DE, and EEL-ABC. In each tested scenario, EEL-GA consistently outperformed in terms of energy efficiency, latency minimization, and network longevity—supporting its suitability for real-world forestry deployments. In future work, the EEL-GA protocol may be extended by integrating lightweight encryption modules to address the security dimension without increasing computational burden. Additionally, the dynamic adjustment of cluster parameters in response to seasonal changes or critical events (e.g., wildfire risk or illegal logging alerts) can be incorporated to improve autonomous response. Field validation in additional forested regions could further support the scalability and adaptability of the model in diverse ecological settings.

## Figures and Tables

**Table 1:** Comparison of energy efficiency and energy harvesting in WSNs.

Aspect	Energy Efficiency (EE)	Energy Harvesting (EH)
**Operational Stability**	Offers consistent performance regardless of external factors [51]	Highly dependent on environmental conditions, like solar or wind availability [48]
**Hardware Requirements**	Lower hardware complexity, based on standard microcontrollers and sensors [52]	Requires additional circuits for MPPT, energy storage, and regulators [52]
**Economic Viability**	Faster return on investment; reduced energy bills and maintenance [53]	Higher upfront cost; cost-effective only in long-term and specific conditions [46]
**Environmental Benefits**	Indirectly reduces carbon footprint through lowered energy use [50]	Offers green energy collection, but effectiveness varies [48]
**Implementation Scalability**	Scalable in urban and remote settings, without reliance on ambient energy [54]	Limited scalability in shaded, enclosed, or indoor deployments [46]
**Suitability for WSNs**	Suitable for mission-critical and delay-sensitive WSN applications [52]	Best suited as a backup energy source, not as a primary power strategy [48]

**Table 2:** WSN deployment optimization techniques.

Author	Year	Objectives	Optimization Method
[56]	2022	Maximize Coverage, Connectivity, Minimize Cost	Gray Wolf Optimization
[57]	2020	Coverage, Cost, Connectivity	NSGA-II
[58]	2020	Coverage, Cost, Connectivity	NSGA-II
[59]	2020	Coverage, Connectivity	GA, PSO
[60]	2019	Coverage, Network Lifetime, Connectivity	NSGA-III
[61]	2019	Coverage, Lifetime, Energy Dissipation, Connectivity	MOPFA
[62]	2018	Lifetime, Cost, Fault Tolerance, Connectivity	Smart Bat Algorithm

**Table 4:** Simulation parameters for EEL-GA in 3D WSN.

Parameter	Value
Number of nodes	200
Network size	1000 m × 1000 m × 1000 m
Base station position	(500, 500, 500) m
Initial energy per node	0.5±0.5 J
Packet size	1000–4000 bits
CH selection probability (*p*)	0.04
Rounds simulated	11 (initial analysis, extendable for long-term evaluation)
GA population size	10
Data aggregation energy ($E_{DA}$)	5 nJ/bit
Tx/Rx energy ($E_{elec}$)	50 nJ/bit
Free space energy ($E_{fs}$)	10 pJ/bit/m^2^
Multipath energy ($E_{mp}$)	0.0013 pJ/bit/m^4^
Environmental conditions	Real dataset
Deployment type	Randomized 3D probabilistic

**Table 5:** Simulation parameters and justifications.

Parameter	Description	Value / Justification
α	Weight for residual energy in fitness	0.6 (empirically tuned)
GA population size	Number of chromosomes	10 (resource-aware trade-off)
Max generations	Number of GA iterations	30 (converged within limit)
CH selection probability *p*	LEACH-based CH percentage	0.05 (standard in WSN)

**Table 6:** Environmental variables and EEL-GA adaptation strategies.

Environmental Factor	WSN Impact	EEL-GA Adaptation
Temperature	Accelerated battery depletion and reduced signal strength [101]	Dynamic adjustment of node duty cycles and transmission power
Humidity	Signal attenuation and increased packet loss [101]	Selection of alternative routing paths and link-quality-based clustering
Precipitation	Elevated risk of node failure and path loss [102]	Activation of redundant nodes and rerouting to maintain connectivity

## Data Availability

The data presented in this study are not publicly available due to institutional or third-party restrictions.
